# The impact of preadmission/prediagnosis use of GLP‐1 receptor agonists on COVID‐19 mortality in patients with diabetes: A systematic review and meta‐analysis

**DOI:** 10.1002/hsr2.1549

**Published:** 2023-09-13

**Authors:** Chia Siang Kow, Dinesh Sangarran Ramachandram, Syed Shahzad Hasan

**Affiliations:** ^1^ Department of Pharmacy Practice School of Pharmacy, International Medical University Kuala Lumpur Malaysia; ^2^ School of Pharmacy Monash University Malaysia Bandar Sunway Selangor Malaysia; ^3^ Department of Pharmacy School of Applied Sciences, University of Huddersfield Huddersfield UK; ^4^ School of Biomedical Sciences & Pharmacy University of Newcastle Callaghan Australia

**Keywords:** dulaglutide, liraglutide, lixisenatide, SARS‐CoV‐2, semaglutide

## 1 INTRODUCTION

From the onset of the coronavirus disease 2019 (COVID‐19) pandemic, there has been considerable interest in exploring the potential of repurposing antidiabetic drugs with anti‐inflammatory properties to improve the outcomes of patients with COVID‐19.^1^ Among these drugs, glucagon‐like peptide‐1 (GLP‐1) receptor agonists have emerged as promising candidates owing to their potential to alleviate inflammation. Previous studies have reported the potential of GLP‐1 receptor agonists to lower the levels of C‐reactive protein and interleukin‐6, both of which have prognostic significance in patients with COVID‐19.^2^, ^3^, ^4^, ^5^, ^6^, ^7^ However, further clinical evidence is needed to fully establish the efficacy of GLP‐1 receptor agonists in this population of patients. Therefore, our objective is to conduct an updated systematic review and meta‐analysis of covariate‐adjusted real‐world studies to evaluate the impact of preadmission/prediagnosis use of GLP‐1 receptor agonists on the risk of mortality in patients with COVID‐19 and diabetes.

## 2 METHODS

This systematic review adhered to the Preferred Reporting Items for Systematic Reviews and Meta‐Analysis (PRISMA) statement.^8^

### 2.1 Literature screening

We conducted a systematic literature search of electronic databases (PubMed, Web of Science, Scopus) and preprint servers (medRxiv, Research Square, SSRN) without any language restrictions, aiming to identify studies that included human subjects. The search spanned from the beginning of available records until May 20, 2023. We employed a comprehensive search strategy using relevant keywords and MeSH terms—“COVID‐19,” “SARS‐CoV‐2,” “GLP,” “glucagon,” “antidiabetic,” and “glucose‐lowering.” Additionally, we manually searched the references of relevant articles for additional studies.

### 2.2 Study selection

The literature screening process was conducted independently by two investigators (CSK and SSH) to identify eligible studies. The inclusion criteria for this systematic review were limited to observational studies that provided information on the risk of COVID‐19‐associated mortality in patients who had used GLP‐1 receptor agonists before COVID‐19 hospital admission or diagnosis, compared to those who had not used them. These studies were required to report adjusted mortality estimates in the form of odds ratio, hazard ratio, or relative risk, along with their corresponding 95% confidence intervals. Excluded from consideration were studies that reported non‐adjusted mortality estimates, as well as comments, case reports, conference papers, animal experiments, letters, and review articles that lacked original data.

### 2.3 Study outcome

The primary outcome of interest was COVID‐19‐associated mortality.

### 2.4 Data extraction

Data extraction was carried out by two investigators (CSK and DSR), who extracted important characteristics from each study. In cases where there were disagreements in the data extraction process, the investigators resolved them through discussion and consensus.

### 2.5 Risk of bias assessment

The methodological quality of the observational studies included in the review was evaluated using the Newcastle‐Ottawa Scale. This scale categorized the studies as low, moderate, or high quality based on assigned scores of 0–5, 6–7, and 8–9, respectively. The assessment of study quality was conducted independently by two investigators (CSK and SSH), who resolved any conflicts through discussion and consensus.

### 2.6 Statistical analysis

The meta‐analysis was conducted using the random‐effects model to estimate the pooled odds ratio of mortality in COVID‐19 patients who received GLP‐1 receptor agonists before admission or diagnosis compared to those who did not. The analysis provided 95% confidence intervals to assess the precision of the findings. Heterogeneity among the included studies was evaluated using *I* ^2^ statistics and the *χ* ^2^ test, with significant heterogeneity defined as *I* ^2^ > 50% and a *p* < 0.10, respectively. All statistical computations were performed using Meta XL, version 5.3 (EpiGear International, Queensland, Australia).

## 3 RESULTS

**Figure 1:**
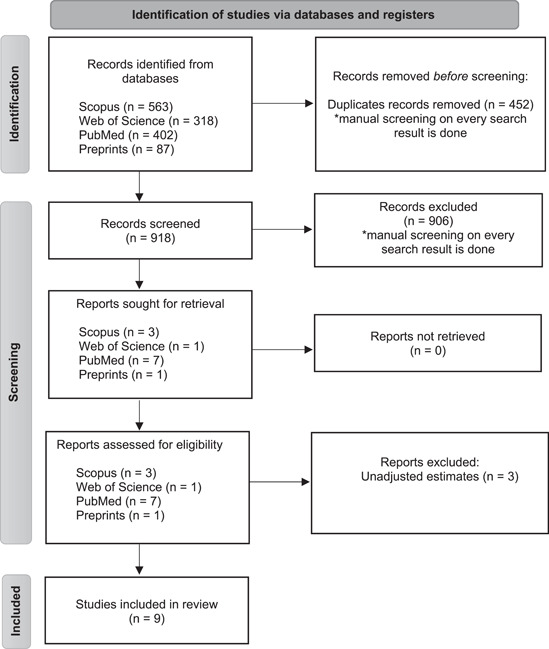
Flow diagram of study selection.

We identified a total of 1370 potential studies through our systematic literature search, and after removing duplicates, 918 unique records remained. Following the screening of titles and abstracts, 12 articles were selected for full‐text review. Figure 1 illustrates the flow diagram of the study selection process. Eventually, nine studies^9^, ^10^, ^11^, ^12^, ^13^, ^14^, ^15^, ^16^, ^17^ met the eligibility criteria and were included in the analysis. Table 1 provides a detailed overview of the characteristics of the included studies.^9^, ^10^, ^11^, ^12^, ^13^, ^14^, ^15^, ^16^, ^17^ Among the nine included studies^9^, ^10^, ^11^, ^12^, ^13^, ^14^, ^15^, ^16^, ^17^ that examined the impact of preadmission/prediagnosis use of GLP‐1 receptor agonists on the risk of mortality in patients with COVID‐19 and diabetes, all of them were retrospective in nature. One study was multicentered,^9^ while the others were database reviews.^10^, ^11^, ^12^, ^13^, ^14^, ^15^, ^16^, ^17^ The quality assessment of the studies, as measured by the Newcastle‐Ottawa Scale, ranged from moderate to high, with scores ranging from 7 to 8 (Table 1).

**Table 1:** Characteristic of included studies.

Study & year	Country	Study design	Total number of patients	Age (median [IQR]/mean [SD])	Mortality			Covariates adjustment	NOS
					GLP‐1 RA users (n/N; %)	Non‐GLP‐1 RA users (n/N; %)	Adjusted estimate (95% CI)		
Wargny et al.^9^ (2020)	France	Retrospective, multicenter	2794	69.7 (13.2)	33/254; 13.0	544/2540; 21.4	OR = 0.78 (0.53–1.15)	Age	8
Israelsen et al.^10^ (2021)	Denmark	Retrospective database review	616	GLP‐1 RA users = 60 (52‐69) Non‐GLP‐1 RA users = 59 (52–68)	14/370; 3.8	9/246; 3.7	RR = 0.89 (0.34–2.33)	Age, sex, duration of glucose‐lowering drug treatment, concomitant use of other glucose‐lowering drugs, diabetic complications, comorbidities, and use of cardiovascular medication	8
Ramos‐Rincón et al.^11^ (2021)	Spain	Retrospective database review	790	Survivors = 85.8 (82.7–88.9) Nonsurvivors = 86.0 (82.7–88.9)	11/24; 45.8	374/766; 48.8	OR = 0.91 (0.50–1.90)	Age, sex, route of acquisition of COVID‐19, body mass index, comorbidities, degree of dependence, Charlson Comorbidity Index, presence pf dyspnea, oxygen saturation <90%, temperature 37.8°C, tachycardia, quick sequential organ failure assessment score ≥2, severity grade of COVID‐19 disease, neutrophils count, lymphocytes count, hemoglobin level, platelet count, glucose level, estimated glomerular filtration rate, lactate dehydrogenase level, c‐reactive protein level, alanine aminotransferase level, use of cardiovascular medications, concomitant use of other glucose‐lowering drugs	7
Nyland et al.^12^ (2021)	Global	Retrospective database review	2326	GLP‐1 RA users = 59.2 (13.6) Non‐GLP‐1 RA users = 64.9 (14.8)	22/1163 (1.9)	38/1163 (3.3)	RR = 0.58 (0.35–0.97)	Age, sex, race, ethnicity, body mass index, comorbidities	8
Wander et al.^13^ (2021)	United States	Retrospective database review	64,892	67.7	N/A	N/A	OR = 0.98 (0.86–1.12)	Age, sex, race/ethnicity, most recent glycated hemoglobin level, prior glucose‐lowering medication use, body mass index, tobacco use, use of an ACE inhibitor, angiotensin receptor blocker, statin, or platelet inhibitor, comorbidities, facility location, month of SARS‐CoV‐2 diagnosis, urban/rural residence by home address	8
Kahkoska et al.^14^ (2021)	United States	Retrospective database review	9650	GLP‐1 RA users = 57.9 (12.6) Non‐GLP‐1 RA users = 59.9 (13.5)	167/6475 (2.6)	169/3175 (5.3)	OR = 0.55 (0.43–0.70)	Age, sex, race/ethnicity, tobacco use, body mass index, bidy weight, glycated hemoglobin level, heart rate, blood pressure, estimated glomerular filtration rate, creatinine level, alanine aminotransferase level, aspartate aminotransferase level, use of cardiovascular and antidiabetic medications, use of remdesivir, comorbidities	8
Ferrannini et al.^15^ (2022)	Sweden	Retrospective database review	2975	GLP‐1 RA users = 76 (67‐83) Non‐GLP‐1 RA users = 66 (58–74)	28/152 (18.4)	974/2823 (34.5)	RR = 0.91 (0.79–1.04)	Age, sex, children, country of birth, income level, education level, living alone, residence in Stockholm, comorbidities, use of cardiovascular medications, concomitant use of other glucose‐lowering drugs	8
Mannucci et al.^16^ (2022)	Italy	Retrospective database review	1923	GLP‐1 RA users = 62.9 All patients = 69.2	10/216 (4.6)	157/1707 (9.2)	OR = 1.33 (0.64–2.77)	Age, sex, concomitant use of other glucose‐lowering drugs, comorbidities	8
Foresta et al.^17^ (2023)	Italy	Retrospective database review	24,850	GLP‐1 RA users = 65.6 (10.1) Non‐GLP‐1 RA users = 72.5 (12.2)	238/1925 (12.4)	482/22,925 (2.1)	OR = 0.91 (0.78–1.06)	Age, sex, duration of diabetes, Drug Derived Complexity Index, comorbidities	8

Abbreviations: CI, confidence interval; COVID‐19, coronavirus disease 2019; GLP‐1 RA, glucagon‐like peptide‐1 receptor agonist; IQR, interquartile range; NOS, Newcastle‐Ottawa Scale; OR, odds ratio; RR, risk ratio; SD, standard deviation

**Figure 2:**
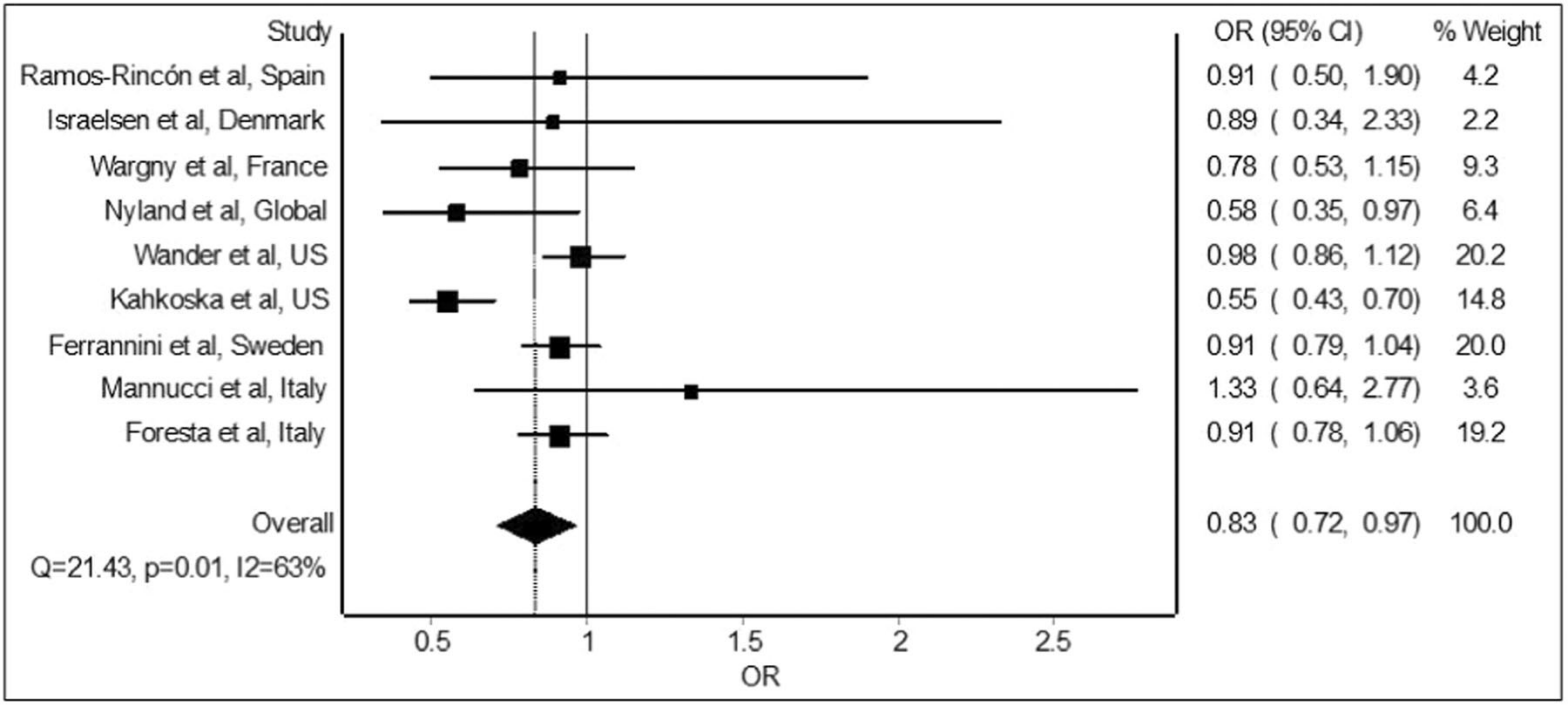
The forest plot displays the pooled odds ratio for all‐cause mortality with the preadmission/prediagnosis use of GLP‐1 receptor agonists relative to non‐use of GLP‐1 receptor agonists in patients with COVID‐19 and diabetes. Each horizontal line represents an individual study, with the square symbol indicating the point estimate of the treatment effect and the horizontal line extending from the square representing the confidence interval. The diamond at the bottom represents the overall summary effect estimate, with its width indicating the confidence interval. Studies favoring the intervention are to the left of the vertical line of no effect, while those favoring the control are to the right. The size of the square indicates the weight of each study in the analysis. Heterogeneity statistics are also included to assess variations in treatment effects across different studies. GLP‐1, glucagon‐like peptide‐1.

The meta‐analysis of the nine studies^9^, ^10^, ^11^, ^12^, ^13^, ^14^, ^15^, ^16^, ^17^ revealed significant reduction in the odds of mortality with preadmission/prediagnosis use of GLP‐1 receptor agonists relative to non‐use of GLP‐1 receptor agonists in COVID‐19 patients with diabetes. The combined analysis of the included studies (Figure 2) shows a pooled odds ratio of 0.83 (95% confidence interval: 0.72–0.97), indicating a beneficial effect of GLP‐1 receptor agonists on mortality.

## 4 DISCUSSION

To our knowledge, this systematic review and meta‐analysis is the first to comprehensively summarize observational studies that have adjusted for covariates and examined the association between the use of GLP‐1 receptor agonists before admission or diagnosis and the risk of mortality in patients with COVID‐19 and diabetes. Our findings revealed significant mortality benefits with the preadmission use of GLP‐1 receptor agonists, which should encourage further investigations through randomized controlled trials.

Some researchers have discussed the potential repurposing of GLP‐1 receptor agonists for the treatment of COVID‐19, citing their ability to exert a pulmonary protective effect by stimulating the angiotensin‐converting enzyme 2 (ACE2)/Angiotensin (1–7)/MasR axis.^18^ Indeed, animal models of lung injury have shown that GLP‐1 receptor agonists can mitigate pulmonary inflammation, reduce cytokine production, and preserve lung function.^19^ Besides, GLP‐1 receptor agonists can exert a favorable influence on gut microbiome composition by enriching *Bacteroidetes*, which is involved in lipopolysaccharide biosynthesis. This effect may play a role in averting the activation of proinflammatory pathways (such as Toll‐Like Receptor 4‐Nuclear Factor Kappa B) due to endotoxemia.^20^ In addition, GLP‐1 receptor agonists can prevent or reduce the sustained hyperglycemia resulting from systemic inflammation related to COVID‐19.^21^

Nevertheless, the use of GLP‐1 receptor agonists is not without risks; GLP‐1‐based therapies have been notoriously linked to gastrointestinal side effects, including nausea, vomiting, and diarrhea, which may complicate gastrointestinal symptoms in patients with COVID‐19.^22^ In addition, the use of GLP‐1 receptor agonists have been infrequently associated with the development of acute kidney injury, particularly in patients with severe gastrointestinal adverse effects. The development of acute kidney injury in patients with COVID‐19 has been associated with an increased risk of mortality.^23^ Consequently, while the potential benefits of GLP‐1 receptor agonists in mitigating the severity of COVID‐19 are intriguing, clinicians must exercise caution and carefully weigh the potential risks, particularly in patients with a predisposition to gatrointestinal issues and renal complications.

It is essential to recognize the inherent limitations of the retrospective design employed in the studies included in our systematic review and meta‐analysis. This design may restrict the generalizability of the findings to a certain extent. Furthermore, our analysis specifically focused on the impact of the use of GLP‐1 receptor agonists before admission or diagnosis in patients with COVID‐19. Therefore, the effects of initiating GLP‐1 receptor agonists as a new treatment in individuals with COVID‐19 cannot be inferred from our analysis. Furthermore, the included studies did not segregate the analysis based on the individual GLP‐1 receptor agonists used, and this specific information was not provided within the scope of the available data. Consequently, we are unable to investigate potential variations introduced by different GLP‐1 receptor agonists.

## 5 CONCLUSION

While the positive findings with the use of GLP‐1 receptor agonists in patients with COVID‐19 and concurrent diabetes are encouraging, clinicians are recommended not to prescribe GLP‐1 receptor agonists solely for the purpose of improving the prognosis of this population of patients before the publication of more solid evidence from randomized controlled trials. Nonetheless, prescribing GLP‐1 receptor agonists amid the COVID‐19 pandemic should not be discouraged, as they can provide both cardioprotective and renoprotective benefits in patients with type 2 diabetes.^24^, ^25^

## AUTHOR CONTRIBUTIONS

**Chia Siang Kow**: Conceptualization; Writing—original draft; Writing—review & editing. **Dinesh Sangarran Ramachandram**: Conceptualization; Writing—review & editing. **Syed Shahzad Hasan**: Writing—original draft; Writing—review & editing.

## CONFLICT OF INTEREST STATEMENT

The authors declare no conflicts of interest.

## TRANSPARENCY STATEMENT

The lead author Chia Siang Kow affirms that this manuscript is an honest, accurate, and transparent account of the study being reported; that no important aspects of the study have been omitted; and that any discrepancies from the study as planned (and, if relevant, registered) have been explained.

## Data Availability

The authors confirm that the data supporting the findings of this study are available within the article.
